# Depth-resolved imaging in turbid media via Mueller matrix polarimetry

**DOI:** 10.1117/1.JBO.30.5.056009

**Published:** 2025-05-15

**Authors:** Xinxian Zhang, Jiahao Fan, Jiawei Song, Nan Zeng, Honghui He, Valery V. Tuchin, Hui Ma

**Affiliations:** a Tsinghua University, Tsinghua Shenzhen International Graduate School, Shenzhen, China; b Nanjing Normal University, School of Teacher Education, Nanjing, China; c Saratov State University, Institute of Physics, Saratov, Russian Federation; d Tsinghua University, Department of Physics, Beijing, China; e Tsinghua University, Tsinghua–Berkeley Shenzhen Institute, Shenzhen, China

**Keywords:** Mueller matrix polarimetry, optical detection, depth sensing, tissue phantoms

## Abstract

**Significance:**

Polarimetry offers advantages such as high information dimensionality and sensitivity to microstructures. Determining the depth of the tissue is essential for clinical diagnosis and treatment, such as lesion localization, removal, and drug delivery. However, relying solely on polarization techniques for tissue depth measurement remains a subject for further investigation.

**Aim:**

We aim to investigate the tissue depth measurement in turbid media using Mueller matrix polarimetry, with a focus on fibrous tissues.

**Approach:**

Tissue phantoms are constructed to quantitatively simulate fibrosis at specific depth. By analyzing Mueller matrix measurements across depth gradients, correlations between polarization basic parameters (PBPs) and tissue depth are established using supervised machine learning algorithms.

**Results:**

We introduce an approach by combining degree of polarization (DOP)-sensitive PBPs with anisotropy-sensitive PBPs to develop depth-sensitive polarization feature parameters (DSPFPs). The DSPFPs exhibit enhanced sensitivity to depth in shallow layers while preserving accuracy in deeper layers. The effectiveness and robustness of the proposed method are validated through 2D depth-resolved imaging of tissue phantoms.

**Conclusions:**

We preliminarily explore the feasibility of depth measurement using Mueller matrix polarimetry, establishing a method for tissue depth assessment while also expanding the applications of polarimetry.

## 1 Introduction

In recent years, optical detection has gained great attention in the biomedical field for its label-free and noninvasive capabilities.^1^^–^^4^ Polarization measurement stands out due to its high sensitivity to the microstructure of biological tissues and its ability to provide higher-dimensional information.^5^^–^^9^ Parameters derived from polarization measurements can offer valuable insights into the morphology, composition, and microstructure of biological tissues, which are essential for pathological diagnosis and tissue classification.^10^^–^^16^ Especially in the detection and diagnosis of skin cancer,^17^^,^^18^ liver cancer,^19^^,^^20^ breast cancer,^10^^,^^21^ colon cancer,^22^^,^^23^ and lung cancer,^24^ polarimetry has been widely applied.

Acquiring depth information of subsurface tissues can provide guidance for lesion removal and drug delivery.^25^^,^^26^ Early studies have demonstrated that polarization techniques can enhance imaging contrast in superficial tissue layers by filtering out multiply scattered photons originating from deeper layers, indicating their potential for depth resolution.^27^^–^^30^ Recent advancements, such as atmospheric dehazing and 3D imaging, suggest that integrating polarization with existing imaging methods can enhance the accuracy of depth extraction.^31^^,^^32^ Combining polarization measurements with depth-sensing technologies allows for the extraction of polarization information at specific depths.^33^^,^^34^ However, achieving depth-resolving capabilities solely through polarimetric measurements remains a challenge that requires further investigation. Preliminary studies by our research group have shown that multi-wavelength polarization techniques can effectively detect layered structures within turbid biological tissues, highlighting the potential of polarimetry for depth measurement.^35^^,^^36^

In this study, we explore the depth-resolving capability of Mueller matrix polarimetry through information extraction. Given the strong scattering properties and structural complexity of biological tissues, tissue phantoms are used to regulate depth and evaluate the effectiveness of depth-resolved imaging.

## 2 Materials and Methods

### 2.1 Experimental Setup and Tissue Phantoms

**Fig. 1:**
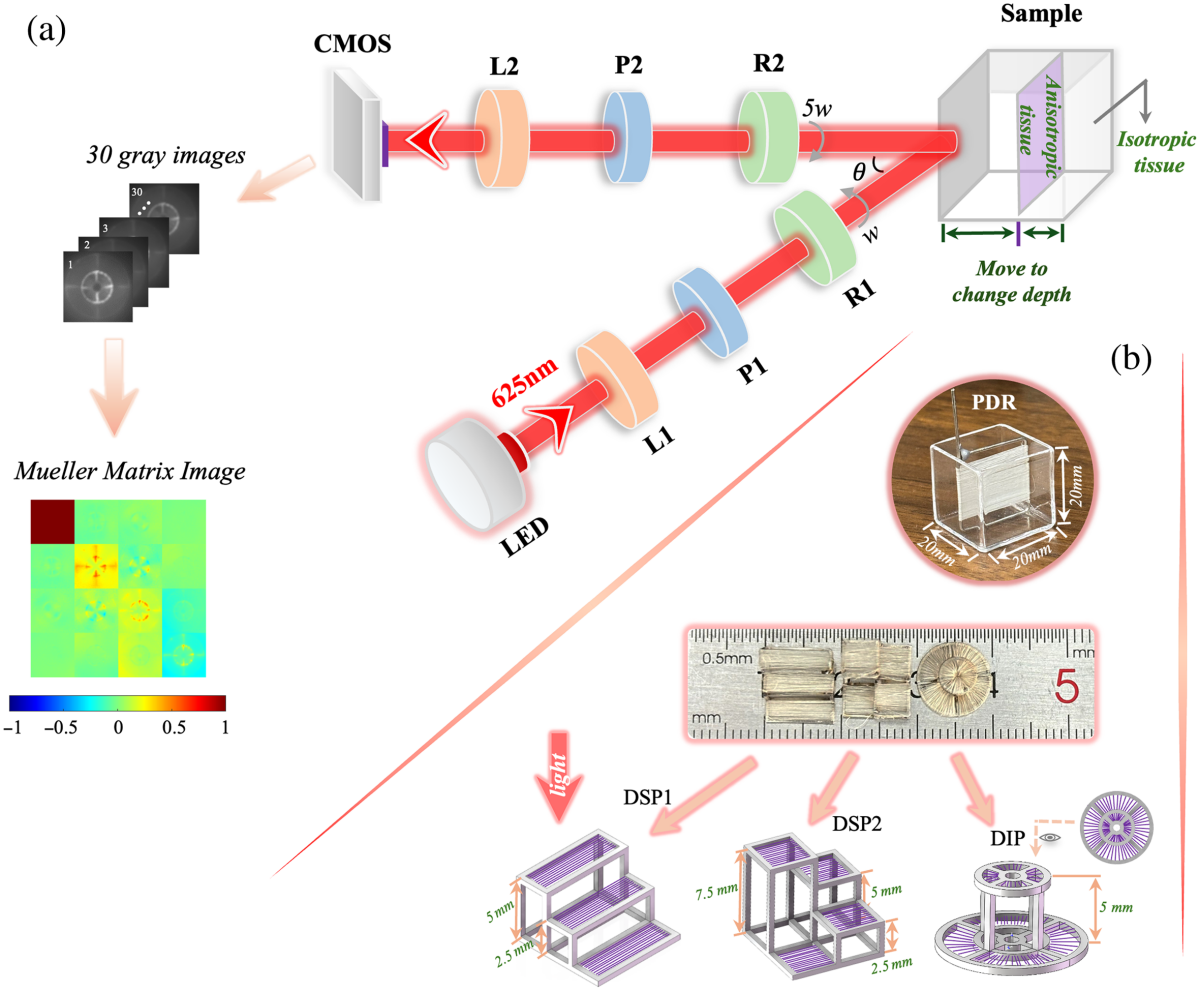
(a) Schematic of the back-scattering Mueller matrix measurement setup and the flowchart of Mueller matrix image computation. (b) Diagram of the tissue phantoms, including the phantom for depth regulation (PDR), direction sensitive phantoms (DSP1, DSP2), and direction insensitive phantom (DIP).

Figure 1(a) shows the experimental setup, which measures the Mueller matrix images by the double-rotating retarder method.^37^ The light beam originates from a broadband LED light source (150W, 400∼1600  nm, Thorlabs OLS2 Fiber Illuminator, United States) and is filtered through a bandpass filter (625 nm, FWHM=10±2  nm, Transmittance >60%, LBTEK MBF10-625, China) to produce monochromatic light. The beam then enters the Polarization State Generator (PSG), where it is modulated to a specific polarization state. The PSG consists of a collimating lens (L1, 400∼700  nm, LBTEK MAD408-A, China), a horizontally transmitting polarizer (P1, 400∼700  nm, LBTEK FLP25-VIS-M), and a quarter-wave plate (R1, 400∼700  nm, LBTEK AQWP25-VIS-A-M, China) that generates various polarization states. After passing through the PSG, the beam is scattered by the sample and then enters the polarization state analyzer (PSA). The PSA includes a quarter-wave plate (R2), a polarizer aligned with the horizontal light transmission direction (P2), and a lens (L2) for focusing parallel light; the models of the components used in the PSA are consistent with those in the PSG. The quarter-wave plates R1 and R2 are driven by rotary motors (Thorlabs PRM1Z8, United States), rotating at fixed angular velocities ω and 5ω, respectively. It is worth noting that the light beam is obliquely incident on the sample surface and is ultimately received frontally by a CMOS camera (12-bit, Hikvision MV-CA016-10UM, China). The angle between the PSG and PSA is set to 15 deg to minimize surface reflections from the sample. Various polarization states are generated by rotating wave plates, and the Mueller matrix image is derived from a collection of 30 grayscale images by calculating the Fourier coefficients.^37^^,^^38^ This Mueller matrix measurement device has been calibrated using air and standard samples, including polarizers and quarter-wave plates, with the systematic error controlled within 1%. Specific calibration details can be found in Ref. 39.

Starting with polarization-sensitive fibrous tissue, the fibrosis phantoms are constructed to regulate the depth quantitatively, in line with the layered characteristics of tissue.^19^^,^^20^ Polystyrene sphere liquid (PSL) is a suspension of polystyrene spheres in water, forming a scattering isotropic turbid medium that resembles the internal environment of biological tissues. Considering that tissue fibrosis typically involves the proliferation of anisotropic fibrous tissues within otherwise isotropic regions, we use a dual-component PSL to simulate the normal tissue environment and silk fibers to mimic fibrotic tissue.^9^^,^^19^^,^^21^^,^^40^^,^^41^ The PSL consists of polystyrene microspheres with diameters of 5 and 1  μm (YUAN BIOTECH, China; Sigma-Aldrich, United States) at volume fractions of 4% and 0.1%, respectively, resulting in a total scattering coefficient of $75.5\;{\mathrm{cm}}^{-1}$. The silk fibers are wound onto a frame to ensure a controlled orientation.

Figure 1(a) presents the conceptual diagram of the phantom for depth regulation (PDR), whereas Fig. 1(b) shows the actual PDR, which consists of a quartz container holding the dual-component PSL and silk wound around a metal frame. By sliding the frame within the PDR along the PSA direction, the target layer’s depth can be adjusted, simulating fibrosis at specific depths. To validate depth-resolved imaging, phantoms with fixed depth gradients, including direction-sensitive phantoms (DSP1 and DSP2) and a direction-insensitive phantom (DIP), are constructed. The DIP is consisted by fibers wound in divergent orientations.^14^ Figure 1(b) shows images of DSP1, DSP2, and DIP. The polarization characteristics of the quartz containers are found to be weak, indicating minimal influence on the measurements.

### 2.2 Polarization Basic Parameter (PBP) Derived from Mueller matrix

Mueller matrix covers complete polarization information of the medium. However, because individual matrix elements lack clear physical meaning and are sensitive to sample orientation, methods such as matrix transformation and decomposition are used to improve interpretability.^5^

The Mueller matrix polar decomposition (MMPD) method proposed by Lu and Chipman is widely used.^42^ Through multiplicative decomposition, the Mueller matrix is expressed as a product of a dichroism matrix, a retardance matrix, and a depolarization matrix. Accordingly, it introduces a series of physical parameters, including diattenuation (D), depolarization (Δ), linear phase retardance (δ), and circular phase retardance (ψ). In addition to multiplicative decomposition, additive decomposition is also commonly used, especially the Mueller matrix Cloude decomposition (MMCD).^43^ This method maps the Mueller matrix into a Hermitian matrix using Pauli matrices and defines a series of polarization-related parameters based on the eigenvalues of the Hermitian matrix. These parameters include polarization purity indices $\left(P_{1},P_{2},P_{3}\right)$, overall purity index ($P_{I}$), depolarization index ($P_{\Delta }$), and polarization entropy (S).

Besides decomposing the Mueller matrix to extract new parameters, the Mueller Matrix transformation (MMT) method directly combines the elements of the Mueller matrix to extract parameters related to the microstructure and optical properties of biological tissues.^14^^,^^44^ Such as anisotropy parameters $\left(t_{1},t_{2},t_{3}\right)$, linear polarization parameters (b), and normalized anisotropy parameters (A).

Combining the elements of the Mueller matrix can also generate rotation invariant parameters (RIP) that reflect the anisotropy of the medium.^10^ Among these, $P_{L}$ and $P_{C}$ represent the degrees of linear and circular polarization, $D_{L}$ and $D_{C}$ reflect linear and circular dichroism, and $q_{L}$ and $r_{L}$ indicate the conversion capabilities between linear and circular polarization states.

In addition, in previous research, we projected the Mueller matrix onto the Poincaré sphere and established a fully polarized stereoscopic representation: the global-polarization Stokes ellipsoid (GPSE), which enhances the intuitiveness of polarization feature reflection and interpretation.^45^ Correspondingly, based on spatial features, GPSE parameter system can be established, including the V parameter that reflects the polarization-maintaining ability (PMA) of the medium, the $D_{†}$ and E parameters that indicate the degree of anisotropy, and the anisotropy parameter RA defined by the phase delay $\varphi_{k}$ for a specific polarization state, along with the parameters RD and RS defined by the variance and skewness based on RA.

These PBPs along with their definitions are summarized in Table 1.

**Table 1:** Definitions of widely used PBPs derived from Mueller matrix.

Mueller matrix polar decomposition (MMPD)	
$M=M_{\Delta }\cdot M_{R}\cdot M_{D}$	The Muller matrix is decomposed into the product of three submatrices
$D=\sqrt{M_{12}^{2}+M_{13}^{2}+M_{14}^{2}}\in [0,1]$	$\Delta =1-\frac{\left|tr(M_{\Delta })-1\right|}{3}\in [0,1]$
$\delta =\cos^{-1}\{\sqrt{[M_{R}(2,2)+M_{R}(3,3){]}^{2}+[M_{R}(3,2)-M_{R}(2,3){]}^{2}}-1\}$	$\psi =\frac{1}{2}\;\tan^{-1}(\frac{M_{R}(3,2)-M_{R}(2,3)}{M_{R}(2,2)-M_{R}(3,3)})$
Mueller matrix Cloude decomposition (MMCD)	
$H(M)=\frac{1}{4}\sum_{i,j=1}^{4}m_{ij}\sigma_{i}⊗\sigma_{j}=\frac{1}{m_{11}}\sum_{i=1}^{4}\lambda_{i}H_{i}$	The Muller matrix is mapped to a Hermitian matrix by the Pauli matrix
$P_{1}=\frac{\lambda_{1}-\lambda_{2}}{m_{11}}$	$P_{2}=\frac{\lambda_{1}+\lambda_{2}-2\lambda_{3}}{m_{11}}$
$P_{3}=\frac{\lambda_{1}+\lambda_{2}+\lambda_{3}-3\lambda_{4}}{m_{11}}$	$P_{I}=\sqrt{\frac{1}{3}(P_{1}^{2}+P_{2}^{2}+P_{3}^{2})}$
$P_{\Delta }=\sqrt{\frac{1}{3}(2P_{1}^{2}+\frac{2}{3}P_{2}^{2}+\frac{1}{3}P_{3}^{2})}$	$S=-\overset{4}{\underset{i=1}{\sum }}(\frac{\lambda_{i}}{m_{11}}\;\log_{4}(\frac{\lambda_{i}}{m_{11}}))$
Mueller matrix transformation (MMT)	
$t_{1}=\frac{1}{2}\sqrt{(m_{22}-m_{33}{)}^{2}+(m_{23}+m_{32}{)}^{2}}$	$t_{2}=\frac{1}{2}\sqrt{(m_{21}+m_{31}{)}^{2}}$
$t_{3}=\frac{1}{2}\sqrt{(m_{42}+m_{43}{)}^{2}}$	$b=\frac{1}{2}(m_{22}+m_{33})$
$A=\frac{2b\cdot t_{1}}{b^{2}+t_{1}^{2}}\in [0,1]$	
Rotation invariant parameters (RIP)	
$P_{L}=\sqrt{m_{21}^{2}+m_{31}^{2}}\in [0,1]$	$P_{C}=m_{41}\in [-1,1]$
$D_{L}=\sqrt{m_{12}^{2}+m_{13}^{2}}\in [0,1]$	$D_{C}=m_{14}\in [-1,1]$
$q_{L}=\sqrt{m_{42}^{2}+m_{43}^{2}}\in [0,1]$	$r_{L}=\sqrt{m_{24}^{2}+m_{34}^{2}}\in [0,1]$
Global-polarization Stokes ellipsoid (GPSE)	
$a,b_{†},c$	The length of the long axis, middle axis, and short axis of the GPSE
$V=\sqrt[{3}]{a\cdot b_{†}\cdot c}\in [0,1]$	$E=\sqrt{1-\frac{b_{†}^{2}+c^{2}}{2a^{2}}}\in [0,1]$
$D_{†}=\frac{3\sqrt{x_{0}^{2}+y_{0}^{2}+z_{0}^{2}}}{a+b_{†}+c}$	$RA=\lim_{n\to \infty }\frac{1}{n}\cdot \sum_{k=1}^{n}\varphi_{k}$
$RD=\lim_{n\to \infty }\frac{1}{n}\cdot \sum_{k=1}^{n}(\varphi_{k}-RA{)}^{2}$	$RS=\underset{n\to \infty }{\lim}\frac{1}{n}\cdot \frac{1}{RD^{3}}\overset{n}{\underset{k=1}{\sum }}(\varphi_{k}-RA{)}^{3}$

### 2.3 Supervised Machine Learning (SML) Algorithms

In this study, SML regression algorithms are utilized to establish the relationship between polarization parameters and the depth of the target tissue. Three commonly used SML algorithms are selected: support vector regression (SVR), k-nearest neighbors (KNN), and polynomial regression (PR).^46^^,^^47^

KNN predicts target values by calculating distances to training samples and averaging the values of the nearest neighbors, making it particularly suitable for small datasets. SVR determines a hyperplane that best fits most data points while employing kernel functions to handle both linear and nonlinear regression tasks, ensuring robustness to outliers. PR introduces polynomial terms into linear regression, effectively capturing complex nonlinear relationships and uncovering intricate patterns within the data.

$$R^{2}=1-\frac{SS_{\mathrm{res}}}{SS_{\mathrm{tot}}}\in [0,1],$$ (1)

RMSE=1n∑i=1n(yi−yi^)2. (2)

During the training process, the dataset is randomly shuffled, and five-fold cross-validation is applied to evaluate model performance. Model performance is primarily evaluated using the statistical metrics $R^{2}$ and root mean squared error (RMSE), which provide comprehensive assessment for both linear and nonlinear models. Among these, $R^{2}$ measures how well the regression model fits the data, as shown in Eq. (1). In this equation, ${\mathrm{SS}}_{\mathrm{res}}$ represents the unexplained variance, whereas ${\mathrm{SS}}_{\mathrm{tot}}$ denotes the total variance of the target variable. A higher $R^{2}$ value, approaching 1, indicates a better fit of the model to the data. RMSE measures the difference between the predicted values and the actual observations, as shown in Eq. (2), where yi−yi^ represents the residuals.

## 3 Results and Discussions

### 3.1 Evaluation of Depth-Sensing Capabilities of Existing PBPs

To simulate fibrosis lesions at varying depths, the silk layer’s position is adjusted from an initial depth of 2 mm in 2 mm increments, up to a maximum of 20 mm. Polarization data are collected at each depth. The 400×400  pixel detection surface is divided into a 20×20 grid, and the Mueller matrices within each grid are averaged. Six repeated experiments yield a total dataset of 24,000 data points.

**Fig. 2:**
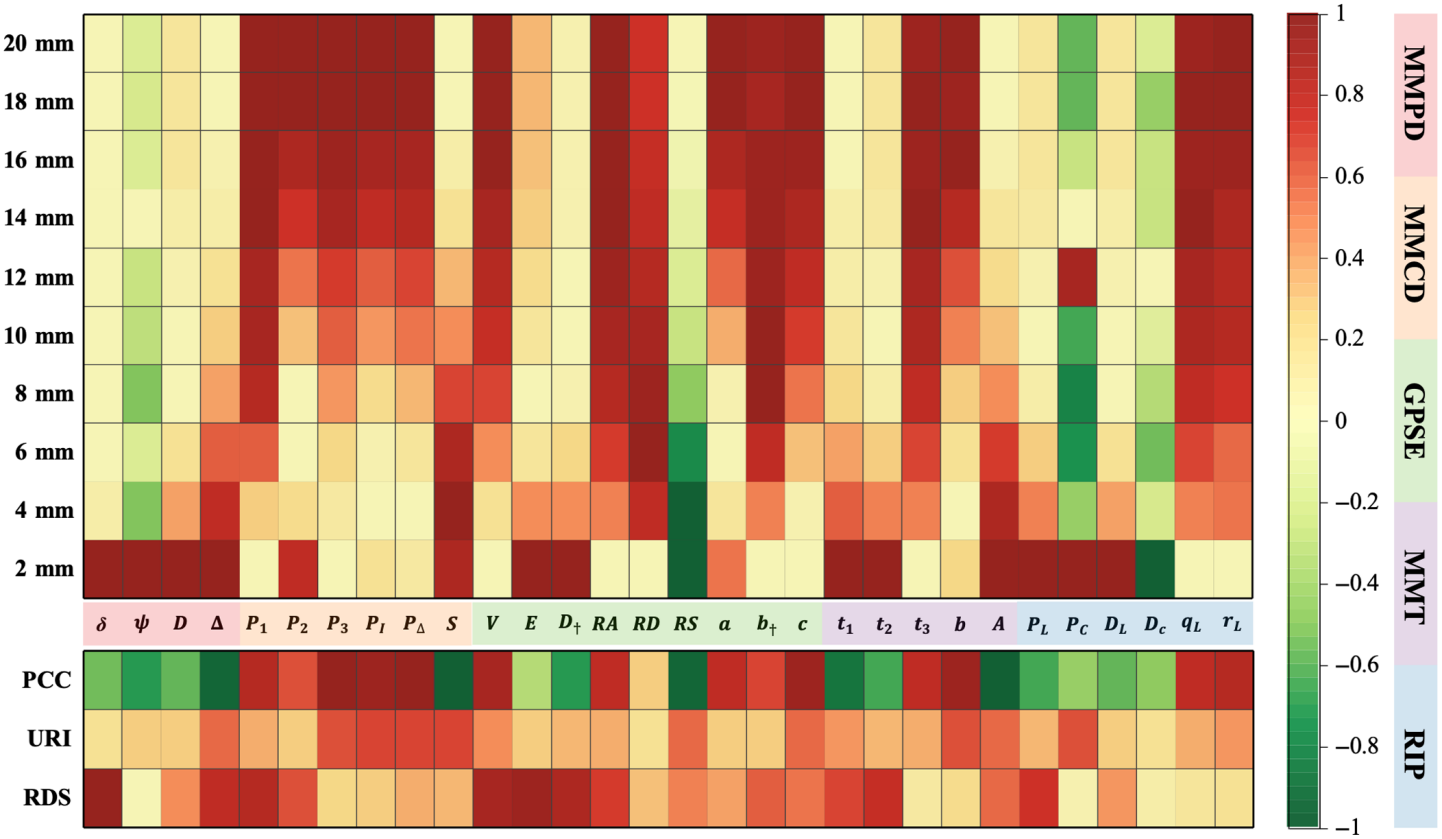
Numerical responses of the PBPs listed in Table 1 to the depth of the fiber layer and the corresponding statistical index.

Previous studies have shown that the polarization properties reflected by PBPs can reveal the microstructure and optical characteristics of biological tissues.^10^ Here, we discuss the numerical responses of the PBPs listed in Table 1 and analyze their depth-sensing capability through statistical indexes of the responses. To facilitate visualization and comparison, the PBPs are presented as a heatmap in Fig. 2, where all elements are normalized to the range of −1 to 1 and displayed using a consistent color bar.

This heatmap can be divided into two parts: the upper half shows the numerical values of PBPs from parameter systems including MMPD, MMCD, GPSE, MMT, and RIP at specific fiber layer depths. The lower half presents statistical metrics of the PBPs’ depth responses, including the Pearson correlation coefficient (PCC), the unsaturated response interval (URI), and the rank of data stability (RDS).

The PCC is used to measure the monotonicity and linear relationship between the numerical response of PBPs and the target tissue depth. The value of PCC ranges from −1 to 1, with values closer to 1 indicating a stronger linear and monotonic response of the PBP to depth. This suggests that the variation of PBP across different depths is more distinct, providing a clearer reflection of depth changes in the tissue. The URI is used to evaluate the sensitivity range of the PBP to depth. When the target layer moves from 2 to 20 mm, if the numerical change of a PBP at a specific depth reaches 80% of the total range, the interval from 2 mm to that depth is defined as the URI. A larger URI indicates that the PBP is sensitive to a wider depth range, reflecting a stronger ability to capture depth information and providing richer depth sensing. The RDS is introduced to assess the stability of the PBP’s response at different depths. RDS is calculated by accumulating the standard deviation of the PBP’s numerical values across various depths, and the PBP’s data stability is ranked based on this accumulation. The RDS is scaled to a range between 0 and 1, with higher RDS values indicating more stable responses and smaller numerical variations across depths.

Based on these three metrics: PCC, URI, and RDS, we aim to select PBPs with high depth sensing capability. Among these metrics, PCC is the most important, as a more monotonic and linear response of the PBP to depth indicates greater potential for depth inversion applications. The second most important metric is URI, as a larger URI shows that the PBP is sensitive to a wider depth range. Although RDS reflects data stability, its relative importance is lower and thus serves as a reference rather than a primary selection criterion.

For example, δ has the highest RDS, indicating strong stability, but its small URI shows limited depth sensitivity. Although δ changes significantly between 2 and 4 mm, its response stabilizes at greater depths, demonstrating poor overall depth-sensing capability.

In the MMPD system, the Δ parameter stands out in terms of PCC, URI, and RDS metrics, indicating excellent depth sensing capability. In the MMCD system, although all PBPs exhibit relatively high PCC values, the depth responses of $P_{2}$, $P_{I}$, and $P_{\Delta }$ are not monotonic, as seen in the upper part of the heatmap. Considering both URI and RDS metrics, $P_{1}$ and S demonstrate particularly strong performance in depth perception. In the GPSE system, the V, $D_{†}$, RA, RS, and c parameters all show high PCC values. Although the URI values of RA and RS are similar, RA has a higher RDS value. Therefore, based on the three statistical metrics, the V, $D_{†}$, R, and c parameters in the GPSE system excel in depth perception. In the MMT system, although all parameters have high PCC values, the depth response of $t_{2}$ and b parameters is not monotonic, and $t_{3}$ shows poor data stability. Conversely, $t_{1}$ and A parameters demonstrate superior depth sensing performance. As for the RIP system, although the $q_{L}$ and $r_{L}$ parameters have excellent PCC values, their low RDS diminishes their effectiveness. $P_{L}$ and $D_{L}$ have moderate RDS values, but the other two metrics are not as favorable. Therefore, the RIP system overall exhibits limited depth perception capability.

Based on the analysis, Δ, P1, S, V, $D_{†}$, RA, c, $t_{1}$, and A demonstrate relatively superior depth-sensing capabilities. Regression models are trained using the three SML algorithms from Sec. 2.3 to retrieve the target layer’s depth based on each of these nine PBPs. The mean absolute error (MAE) is calculated to evaluate retrieval performance at specific depths, whereas the overall performance across the detection range is assessed using $R^{2}$ and RMSE.

**Fig. 3:**
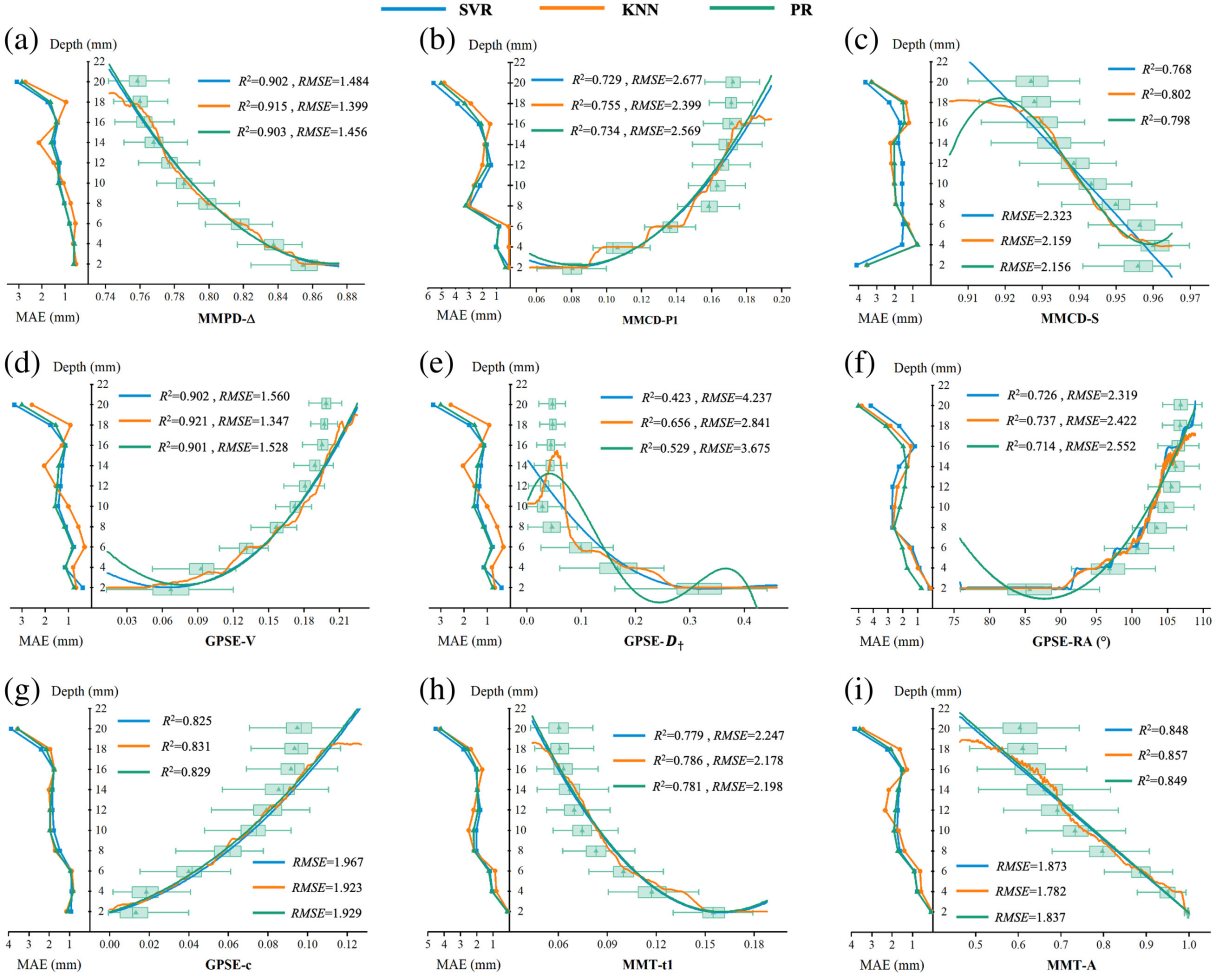
Data distribution of PBPs at specific depths, and depth retrieval results based on SVR, KNN, and PR algorithms (right curve), along with MAE corresponding to the real depths (left curve).

Figure 3 shows the responses of the nine selected PBPs to target layer depth and the model’s retrieval results. Each subplot includes a box plot on the right displaying PBP distributions, with curves on the left showing retrieval errors and curves on the right depicting the retrieved depth values. The nine PBPs in Fig. 3 can be categorized into degree of polarization (DOP)-sensitive type and anisotropy-sensitive type. Among them, Δ, S, P1, V, and c parameters reflect DOP information, whereas $D_{†}$, RA, $t_{1}$, and A parameters reflect the anisotropy information.

Among the five DOP-sensitive PBPs, models based on Δ and V exhibit the best depth retrieval performance, with $R^{2}$ values exceeding 0.9 and RMSE below 1.6 mm across all three SML algorithms. Among the four anisotropy-sensitive parameters, A demonstrates the highest linearity in response to depth, resulting in superior model performance. By contrast, models based on parameter $D_{†}$ yield the highest RMSE and lowest $R^{2}$ values. However, they perform well in retrieving shallow depths, particularly with the KNN algorithm, which achieves an MAE of less than 1 mm for depths under 10 mm. Overall, when the fiber layer depth exceeds 16 mm, the MAE for depth retrieval increases rapidly. This trend suggests that the effective depth range of this optical system is likely within 16 mm.

Figure 3 illustrates that regression models based on anisotropy-sensitive parameters generally outperform those based on DOP-sensitive parameters at shallow depths. However, as the fiber layer depth increases, anisotropy models degrade more rapidly than DOP models. This decline occurs due to stronger depolarization and greater anisotropy in silk tissue compared with the external PSL, resulting in fewer photons reaching the fibrous tissue at greater depths. Further, we can infer that as the fiber tissue moves deeper, polarization information diminishes more gradually, whereas anisotropy information decays at a faster rate.

### 3.2 Development of Depth Feature Parameters

In anomaly detection, classification and localization rely on identifying differences between normal and abnormal tissues. Near the surface, fibrous tissues exhibit distinct characteristics compared with surrounding isotropic tissues. However, as the target layer depth increases, fewer photons carrying information about the target layer reach the detector, making it increasingly difficult to retrieve depth information.

As discussed earlier, models trained on DOP-sensitive parameters perform better across the overall depth range, whereas those based on anisotropy-sensitive parameters excel at shallow depths. Among these, parameter A shows the highest linearity with depth, significantly enhancing model performance. Notably, A is a composite parameter obtained by normalizing $t_{1}$ with b, a anisotropic parameter closely linked to linear DOP. This indicates that combining anisotropy with polarization information could yield superior performance, leveraging the strengths of both anisotropy-sensitive and DOP-sensitive PBPs across different depths.^10^^,^^48^^,^^49^

When the depth of the fibrous layer increases, the PMA of linearly polarized light exhibits different trends depending on whether its vibration direction aligns with or is orthogonal to the fiber orientation. This results in a non-monotonic behavior of the overall linear DOP. By contrast, the circular DOP remains unaffected by this phenomenon. The $m_{44}$ element of the Mueller matrix, which represents the circular DOP of light, is defined by its absolute value as Kc. Multiplicative decomposition preserves the data distribution characteristics. By combining Kc with the PBPs presented in Fig. 3 through basic multiplicative operations, four depth-sensitive polarization feature parameters (DSPFPs), as defined in Eqs. (3)–(6), can be obtained.

$$\mathrm{Ct}=\frac{Kc^{2}}{t_{1}},$$ (3)

$$\mathrm{Ct}\Delta =\frac{Kc}{t_{1}\cdot \Delta },$$ (4)

RV=RA·V, (5)

$$RVD=\frac{RA\cdot V}{1-D_{⏧}}.$$ (6)

The Ct parameter is composed of Kc and $t_{1}$, where Kc represents the circular polarization-maintaining ability (PMA) of the tissue, whereas $t_{1}$ is associated with birefringence. Because Kc and $t_{1}$ respond oppositely to the depth, the reciprocal of $t_{1}$ is multiplied to ensure monotonicity consistency. The parameter Ct is composed of Kc and $t_{1}$, where Kc represents the circular PMA of the tissue, and $t_{1}$ is associated with birefringence. To ensure monotonicity consistency, the reciprocal of $t_{1}$ is used, as Kc and $t_{1}$ exhibit opposing responses to depth. The parameter CtΔ adds the parameter Δ to Ct, with Δ representing the tissue’s overall depolarization across all polarization states. In the RV parameter (converted to radians), RA is linked to tissue birefringence, and V represents the tissue’s PMA across all polarization states. Because both exhibit the same monotonicity, they are directly multiplied. The RVD parameter, based on RV, incorporates the $D_{†}$ parameter to account for tissue dichroism. Given its distribution characteristics, $D_{†}$ is transformed into $1/(1-D_{†})$ to multiplicate with other PBPs.

**Fig. 4:**
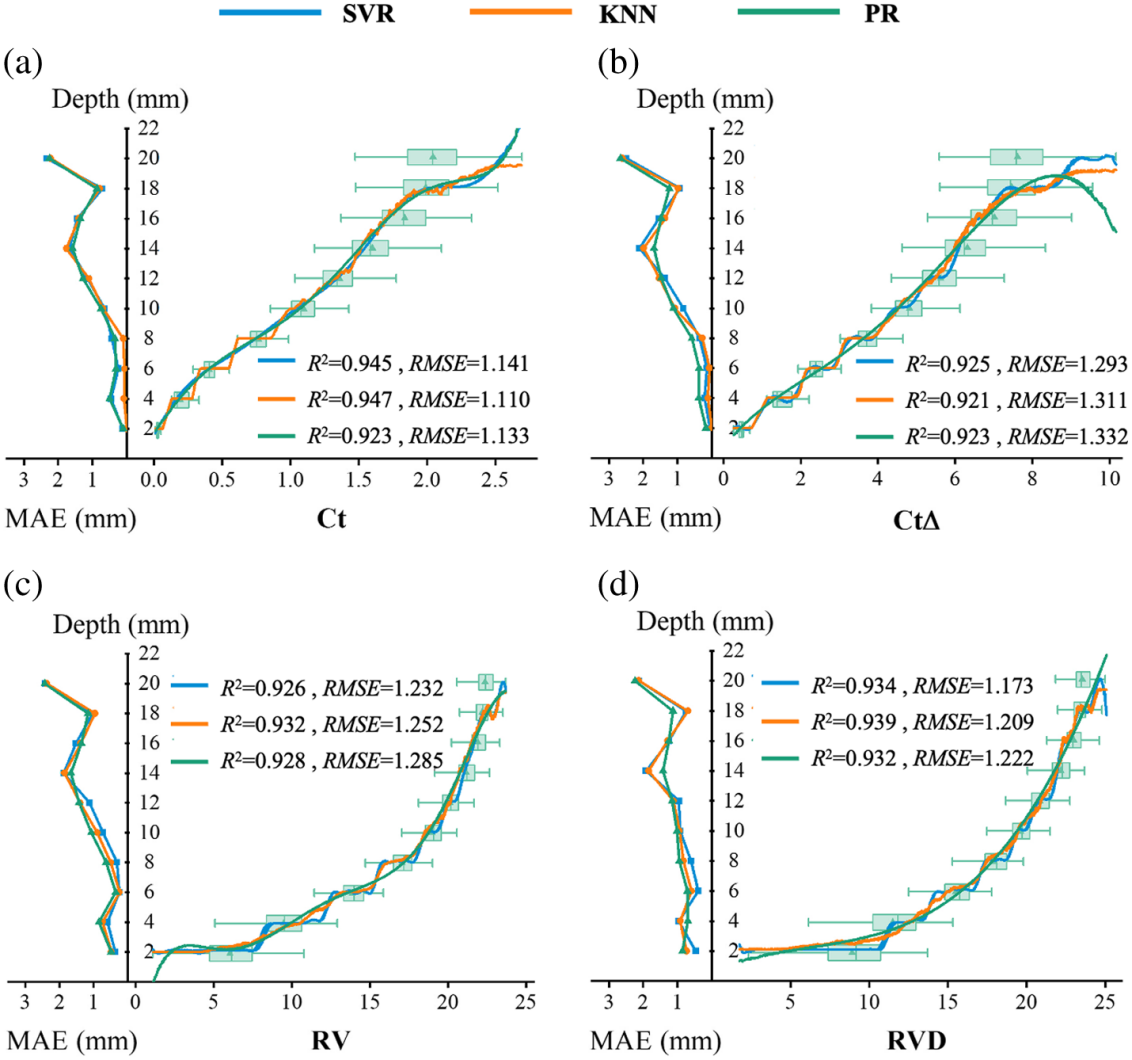
Data distribution of DSPFPs at specific depths, and depth retrieval results based on SVR, KNN, and PR algorithms (right curve), along with MAE corresponding to the real depths (left curve).

Here, we also train regression models using these four DSPFPs. Figure 4 shows the data distribution of these DSPFPs at specific depths, along with the model’s retrieval results and MAE for fiber layer depth. All four DSPFPs exhibit a positive correlation with fiber layer depth, with CtΔ showing the highest linearity. This indicates that even without training regression models, the relative depth of the target layer can be determined semi-quantitatively.

Furthermore, regression models trained based on Ct, CtΔ, and RVD limit the depth retrieval error within ∼1  mm in the shallow layer (2∼10  mm), significantly outperforming models trained based on PBPs listed in Table 1. When the fibrous tissue is in deep layer (10  mm∼16  mm), the retrieval error of the model does not exceed 3 mm, still surpassing the ability of PBPs. Regardless of the SML algorithm used, models based on DSPFPs achieve $R^{2}$ values exceeding 0.92 and an RMSE below 1.34 mm, outperforming all models presented in Fig. 3. This demonstrates that combining PBPs to create DSPFPs is effective, preserving the depth sensitivity of anisotropy-sensitive PBPs in shallow layers while maintaining robust depth perception in deeper layers.

Compared with the models trained based on multiple input parameters, the performance of the models referred to in Figs. 3 and 4 still has limitations. Here, we retrained regression models using parameter groups of the same type: DOP-sensitive PBPs, anisotropy-sensitive PBPs, and DSPFPs. Based on $R^{2}$ and RMSE performance of the models, we selected Δ, V, and c from DOP-sensitive PBPs to train model $M_{p}$; $D_{†}$, RA, and $t_{1}$ from anisotropy-sensitive PBPs for model $M_{A}$ (A, being a composite parameter, was excluded); and Ct, CtΔ, and RVD from DSPFPs for model $M_{D}$. Table 2 lists the MAE for each model at specific depths, whereas $R^{2}$ and RMSE are used to evaluate the overall model performance.

**Table 2:** MAE of regression models trained using SVR, KNN, and PR algorithms, based on DOP-sensitive PBPs, anisotropy-sensitive PBPs, and DSPFPs, at specific fibrous tissue depths, along with RMSE and $R^{2}$ of the models. The units of MAE and RMSE are mm.

	$M_{p}$			$M_{A}$			$M_{D}$		
	SVR	KNN	PR	SVR	KNN	PR	SVR	KNN	PR
2 mm	0.44	0.17	0.20	0.10	0.09	0.67	0.07	0.09	0.14
4 mm	0.58	0.15	0.29	0.54	0.73	0.90	0.17	0.22	0.38
6 mm	0.67	0.14	0.38	0.53	1.00	0.91	0.13	0.10	0.29
8 mm	0.88	0.37	0.43	1.46	2.50	1.81	0.16	0.14	0.40
10 mm	1.15	0.74	0.42	1.65	2.29	1.76	0.48	0.55	0.57
12 mm	1.17	1.11	0.78	1.86	2.09	1.79	0.66	0.69	0.79
14 mm	1.29	1.69	0.91	2.19	1.91	1.86	1.48	1.34	1.38
16 mm	1.14	1.29	0.95	1.63	1.03	1.54	0.95	1.02	1.00
18 mm	1.54	0.74	0.83	1.69	1.74	1.86	0.65	0.73	0.77
20 mm	2.94	2.08	1.44	3.23	3.67	3.54	1.69	1.65	1.63
**RMSE**	1.358	1.067	0.757	1.721	1.965	1.831	0.843	0.835	0.867
$R^{2}$	0.921	0.949	0.974	0.846	0.812	0.841	0.968	0.969	0.967

Among models trained with the same SML algorithms, model $M_{D}$ shows a smaller MAE for depth retrieval in shallow layers (2 to 10 mm), indicating that model $M_{D}$ not only retain depth sensitivity from anisotropy information but also outperform model $M_{A}$. In deeper layers (10 to 16 mm), $M_{D}$ continues to excel compared with its counterparts. Overall, when evaluating model performance across the entire depth detection range using RMSE and $R^{2}$, the ranking for depth retrieval capability is $M_{D}>M_{p}>M_{A}$.

In conclusion, whether trained on single or multiple parameters, models utilizing DSPFPs demonstrate superior depth retrieval compared with those based on anisotropy-sensitive or DOP-sensitive PBPs. This confirms that integrating polarization and anisotropy information to extract DSPFPs is an effective approach.

### 3.3 Evaluation of Depth-Resolved Imaging Performance

In previous sections, we preliminarily examined the depth-sensing capabilities of PBPs and DSPFPs. To further validate the depth-resolved imaging performance, we design tissue phantoms with fixed depth gradients, capable of distinguishing test areas at varying depths, as illustrated in Fig. 1(b). These phantoms are constructed using metal frames positioned at different depths, wrapped in silk, and immersed in the same dual-component PSL used in PDR experiments.

After performing Mueller matrix imaging on these phantoms, depth images can be calculated using the regression models in Sec. 3.2. The top-performing models are selected based on their RMSE and $R^{2}$ values: $M_{p}$ trained with the PR algorithm, $M_{A}$ with the SVR algorithm, and $M_{D}$ with the KNN algorithm. In addition, the Mueller matrix provides a fundamental representation of polarization characteristics, serving as the foundation for the development of both PBPs and DSPFPs. To evaluate the depth imaging performance of models trained directly on the Mueller matrix compared with those utilizing derived polarization parameters, an additional regression model, $M_{M}$, is introduced. This model is trained using the SVR algorithm based on the $m_{44}$ element, which exhibits the strongest depth-sensing capability within the Mueller matrix.

**Fig. 5:**
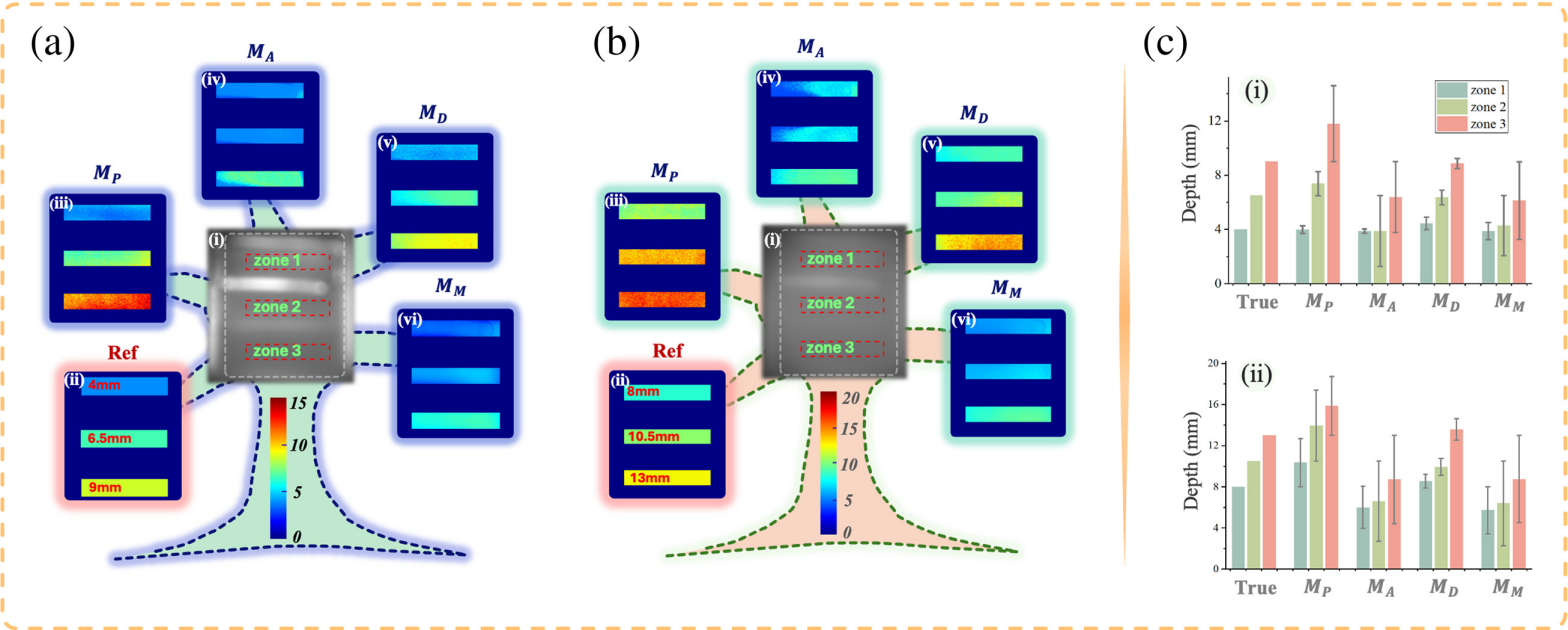
Depth-resolved imaging results for DSP1, with zone 1 located at depths of 4 mm in panel (a) and 8 mm in panel (b). For both panels (a) and (b): (i) grayscale imaging results; (ii) actual depth reference; (iii)–(vi) depth-resolved images from $M_{p}$, $M_{A}$, $M_{D}$, and $M_{M}$ models, respectively. In panel (c), (i) and (ii) show the actual depths from panels (a) and (b), and the depth mean values with MAE (error bars) from the four models.

Figure 5(a,i) presents the grayscale imaging results of DSP1, where strong reflections from the metal frames are evident, potentially interfering with the polarization measurements of adjacent tissues. To address this, we concentrate on the region of interest outlined by the red dashed box. This region is divided into three zones corresponding to fiber layers at varying depths, with a 2.5-mm depth gradient between each zone. Once the actual depth of zone 1 is established, the actual depths of the remaining zones can be determined.

When DSP1 is positioned at shallow depths (4 to 9 mm), the depth images calculated by the four models are presented in Figs. 5(a,iii)–5(a,vi). The true depth is visually represented in Fig. 5(a,ii). When target layer locates at 4-mm depth, all models show accurate results, with mean MAEs under 0.5 mm. Especially, model $M_{A}$, trained on anisotropy-sensitive PBPs, performs best, although its MAE reaches 2.61 mm at 6.5 mm depth. When target layer locates at 9 mm, results of $M_{D}$ remain accurate, whereas $M_{P}$ tends to overestimate depth. For deeper depths (8 to 13 mm), shown in Fig. 5(b), $M_{D}$ continues to yield accurate results, whereas other models show more significant errors. For fibers at 13 mm, MAEs for models $M_{A}$ and $M_{M}$ exceed 4 mm.

Overall, model $M_{D}$ maintains high accuracy at both shallow and deep depths, whereas $M_{A}$ is only reliable at shallow depths. The model $M_{M}$, based on $m_{44}$, performs poorly, and $M_{p}$ has moderate accuracy. These findings are consistent with earlier observations that anisotropy-sensitive PBPs lose depth sensitivity at greater depths, whereas DOP-sensitive PBPs provide enduring depth information. DSPFPs effectively combine both strengths, enhancing depth perception. The results demonstrate that Mueller matrix measurements can resolve depth where light intensity fails. Furthermore, PBPs derived from the Mueller matrix enhance depth sensing accuracy, whereas DSPFPs further improve performance, achieving superior precision.

**Fig. 6:**
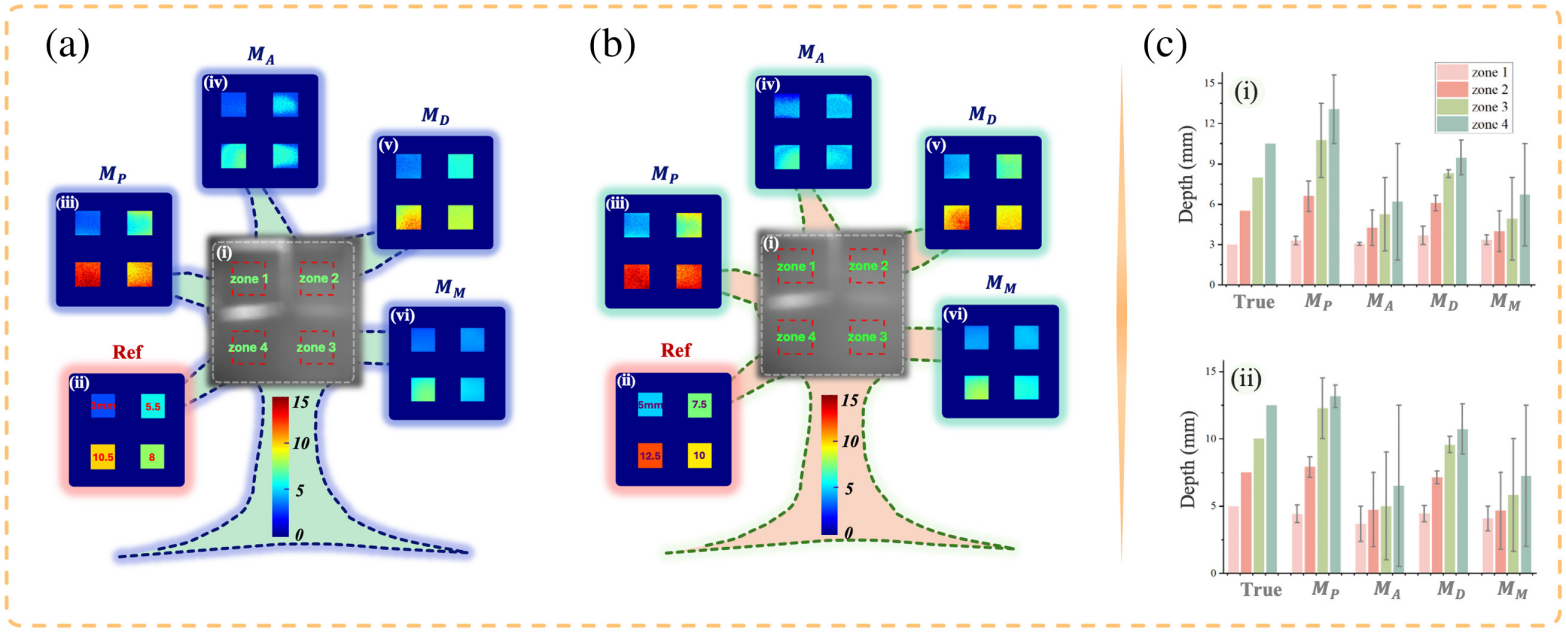
Depth-resolved imaging results for DSP2, with zone 1 located at depths of 3 mm in panel (a) and 5 mm in panel (b). In panel (c), (i) and (ii) show the actual depths from panels (a) and (b), and the depth mean values with MAE (error bars) from the four models.

DSP2 includes an additional zone compared with DSP1, totaling four diagonally arranged zones with a 2.5-mm depth gradient. By positioning zone 1 of DSP2 at depths of 3 and 5 mm, we can assess depth imaging performance in the ranges of 3 to 10.5 mm and 5 to 12.5 mm, as shown in Figs. 6(a) and 6(b).

In Fig. 6(a), we can observe model $M_{D}$ consistently delivers the most accurate depth imaging. At the depth of 10.5 mm, $M_{D}$ achieves an MAE of 1.30 mm, whereas $M_{A}$, $M_{M}$, and $M_{P}$ have MAEs of 4.33, 3.81, and 2.55 mm, respectively. Figure 6(b) also supports this finding: $M_{D}$ maintains good accuracy at deeper layers, whereas $M_{A}$ and $M_{M}$ show significant accuracy loss and $M_{P}$ performs moderately.

Considering the significant impact of sample orientation on polarization measurements, particularly for anisotropic fibrous tissues, DIP is designed to evaluate its influence on imaging results.^14^ This phantom consists of two circular metal frames positioned at different depths, with silk fibers radially wrapped around the frames.

**Fig. 7:**
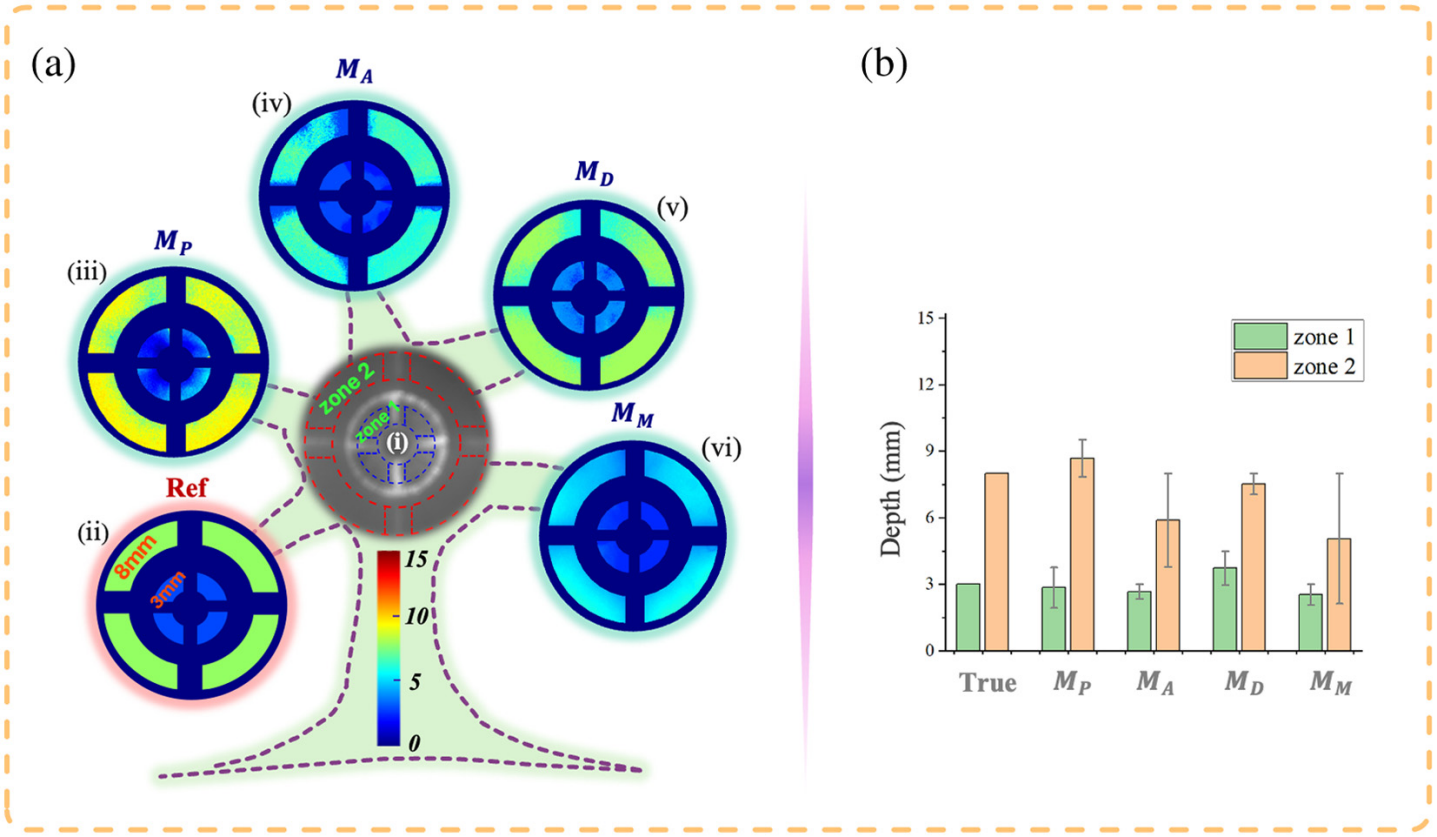
Depth-resolved imaging results for DIP, with zone 1 located at depths of 3 mm. (b) The actual depths from panel (a), and the depth mean values with MAE (error bars) from the four models.

Figure 7 displays the depth imaging results for DIP. In Fig. 7(a,i), the blue box (zone 1) represents a depth of 3 mm, whereas the red box (zone 2) indicates 8 mm. Despite reflections at the metal frame intersections, the color consistency within each layer suggests that the depth imaging method is insensitive to the sample orientation. In fact, the polarization parameters used to train the models are rotation-invariant, supporting this conclusion.

## 4 Conclusions

This study employs Mueller matrix polarimetry to sense the depth of fibrous tissue in turbid media and validates the effectiveness of depth-resolved imaging. To enable depth regulation of fibrous tissue, tissue phantoms were designed, including the PDR, which consists of layered silk fibers to simulate anisotropic fibrous tissue, and a dual-component PSL to represent isotropic tissue. By adjusting the target layer depth and measuring the Mueller matrices, PBPs from MMPD, MMCD, GPSE, MMT, and RIP methods were computed. Experimental results indicate that anisotropy-sensitive PBPs exhibit strong depth responsiveness at shallow layers but deteriorate rapidly with increasing depth, whereas DOP-sensitive PBPs provide more consistent depth sensing. To leverage the advantages of both types of PBPs, four DSPFPs were introduced, significantly enhancing depth-sensing accuracy. Furthermore, the depth-resolved imaging performance and robustness were evaluated using tissue phantoms with fixed depths, including unidirectionally oriented DSP1 and DSP2, as well as the divergently oriented DIP. The results demonstrate that polarization-based measurements can effectively differentiate depths where grayscale imaging fails. Compared with direct analysis of Mueller matrix elements, PBPs derived from the Mueller matrix improve depth sensing capability, whereas DSPFPs further enhance this capability. Overall, this study establishes the feasibility of depth sensing using Mueller matrix polarimetry and presents a methodology for localizing fibrous tissue depth in turbid media. The approach holds promise for clinical applications, including lesion localization, excision, and targeted drug delivery.

## Data Availability

Data underlying the results presented in this paper are not publicly available at this time but may be obtained from the authors upon reasonable request.
